# RETRACTION: Guanylate‐Binding Protein 1 Correlates With Advanced Tumor Features and Serves as a Prognostic Biomarker for Worse Survival in Lung Adenocarcinoma Patients

**DOI:** 10.1002/jcla.70238

**Published:** 2026-04-13

## Abstract

**RETRACTION**: Q. Wan, J. Qu, L. Li, F. Gao, “Guanylate‐Binding Protein 1 Correlates With Advanced Tumor Features and Serves as a Prognostic Biomarker for Worse Survival in Lung Adenocarcinoma Patients,” *Journal of Clinical and Laboratory Analysis* 35, no. 2 (2020): e23610, https://doi.org/10.1002/jcla.23610.

The above article, published online on 10 December 2020 in Wiley Online Library (wileyonlinelibrary.com), has been retracted by agreement between the journal Editor‐in‐Chief, Rong Fu; and Wiley Periodicals, LLC. The retraction has been agreed due to concerns raised by a third party, which identified compromised data and systematic issues in the published article, and several flaws and inconsistencies between results presented and experimental methods described were found. Furthermore, several claims lack sufficient support from literature. The authors and their institutes were unresponsive when these concerns were brought to their attention. Accordingly, the article is retracted as the editors have lost trust in the reliability of the data and the results presented.

**RETRACTION**: Q. Wan, J. Qu, L. Li, F. Gao, “Guanylate‐Binding Protein 1 Correlates With Advanced Tumor Features and Serves as a Prognostic Biomarker for Worse Survival in Lung Adenocarcinoma Patients,” *Journal of Clinical and Laboratory Analysis* 35, no. 2 (2020): e23610, https://doi.org/10.1002/jcla.23610.

The above article, published online on 10 December 2020 in Wiley Online Library (wileyonlinelibrary.com), has been retracted by agreement between the journal Editor‐in‐Chief, Rong Fu; and Wiley Periodicals, LLC. The retraction has been agreed due to concerns raised by a third party, which identified compromised data and systematic issues in the published article, and several flaws and inconsistencies between results presented and experimental methods described were found. Furthermore, several claims lack sufficient support from literature. The authors and their institutes were unresponsive when these concerns were brought to their attention. Accordingly, the article is retracted as the editors have lost trust in the reliability of the data and the results presented.

